# Supplementary material for: Mechanosensitive channel YnaI has lipid-bound extended sensor paddles
Source: Commun Biol. 2021 May 20;4:602. doi: 10.1038/s42003-021-02122-0 (PMC8137935; doi:10.1038/s42003-021-02122-0)

**Figure S1. Purification of YnaI. a,** Gel filtration of YnaI in LMNG using a Superose 6 column. **b,** SDS-PAGE analysis of the peak fractions from a.

**a**

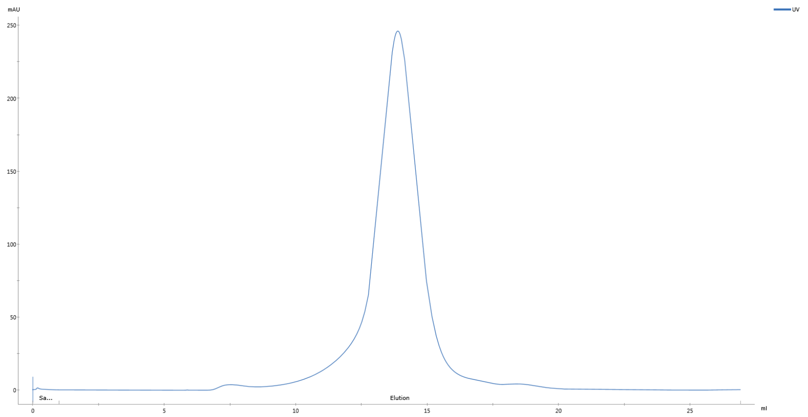


**b**

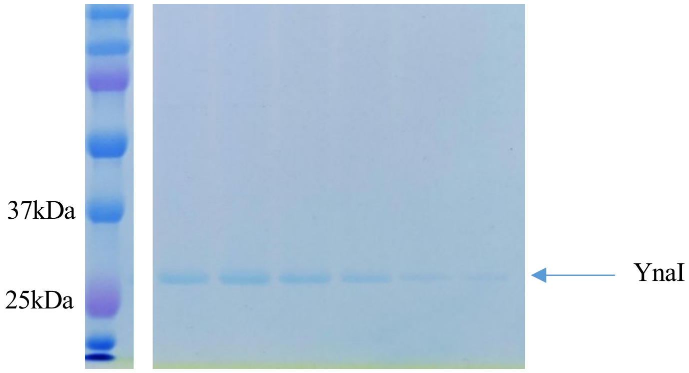

**Figure S2. Negative stain analysis of YnaI in LMNG. a,** A representative negative stain image of YnaI with a scale bar of 200nm. **b,** 2D averages of the negatively stained YnaI particles.

a

b

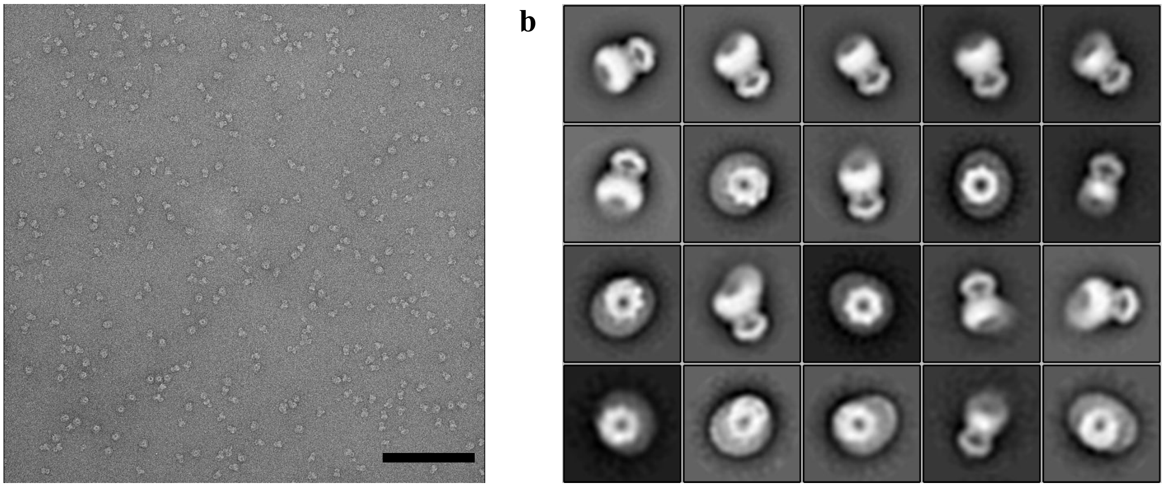

**Figure S3. Cryo-EM analysis of YnaI. a & b,** A representative cryo-EM image of YnaI in LMNG and its corresponding 2D averages mostly in top/bottom views. **c & d,** A representative cryo-EM image of YnaI in LMNG with fluorinated fos-choline 8 and its corresponding 2D averages with evenly distributed views.

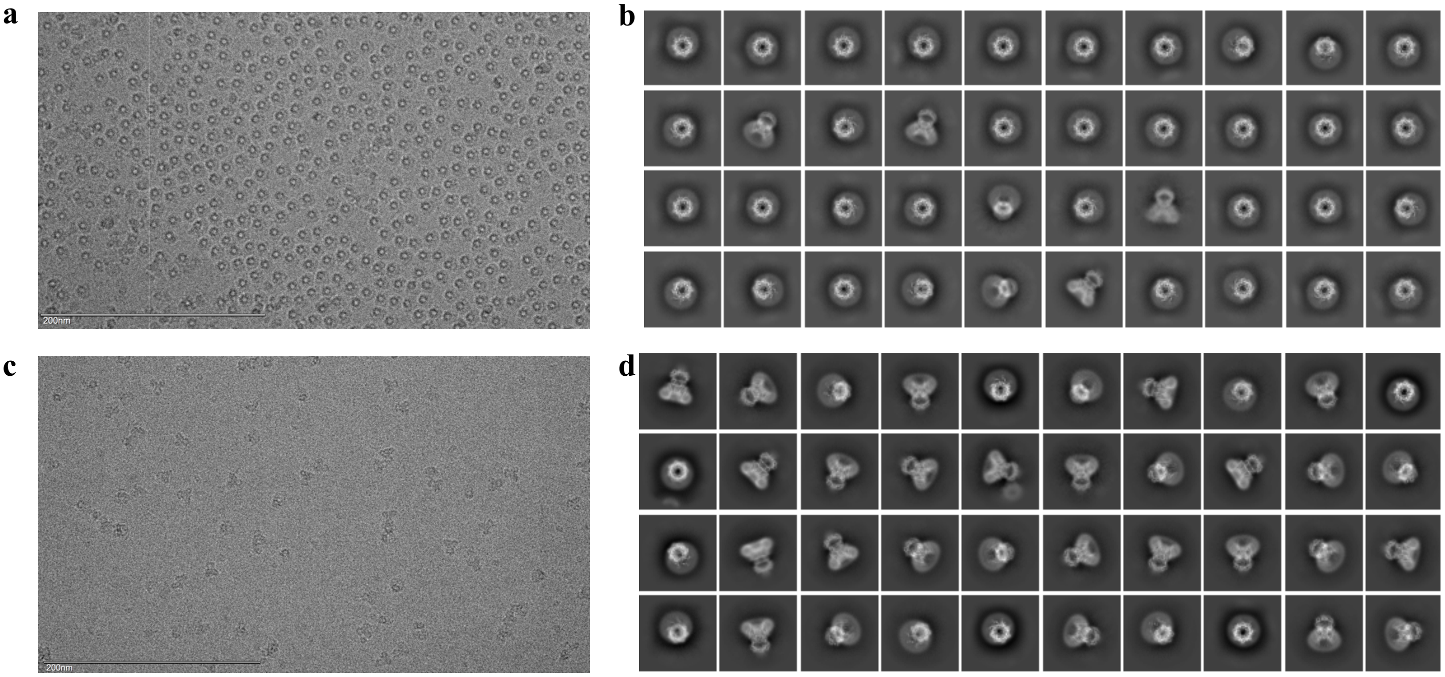

**Figure S4. Resolution estimation of the final YnaI reconstruction. a,** FSC curves from RELION indicate the overall resolution of the final reconstruction at 3.3Å. **b,** Side and top views of the YnaI reconstruction colored by local resolution estimation.

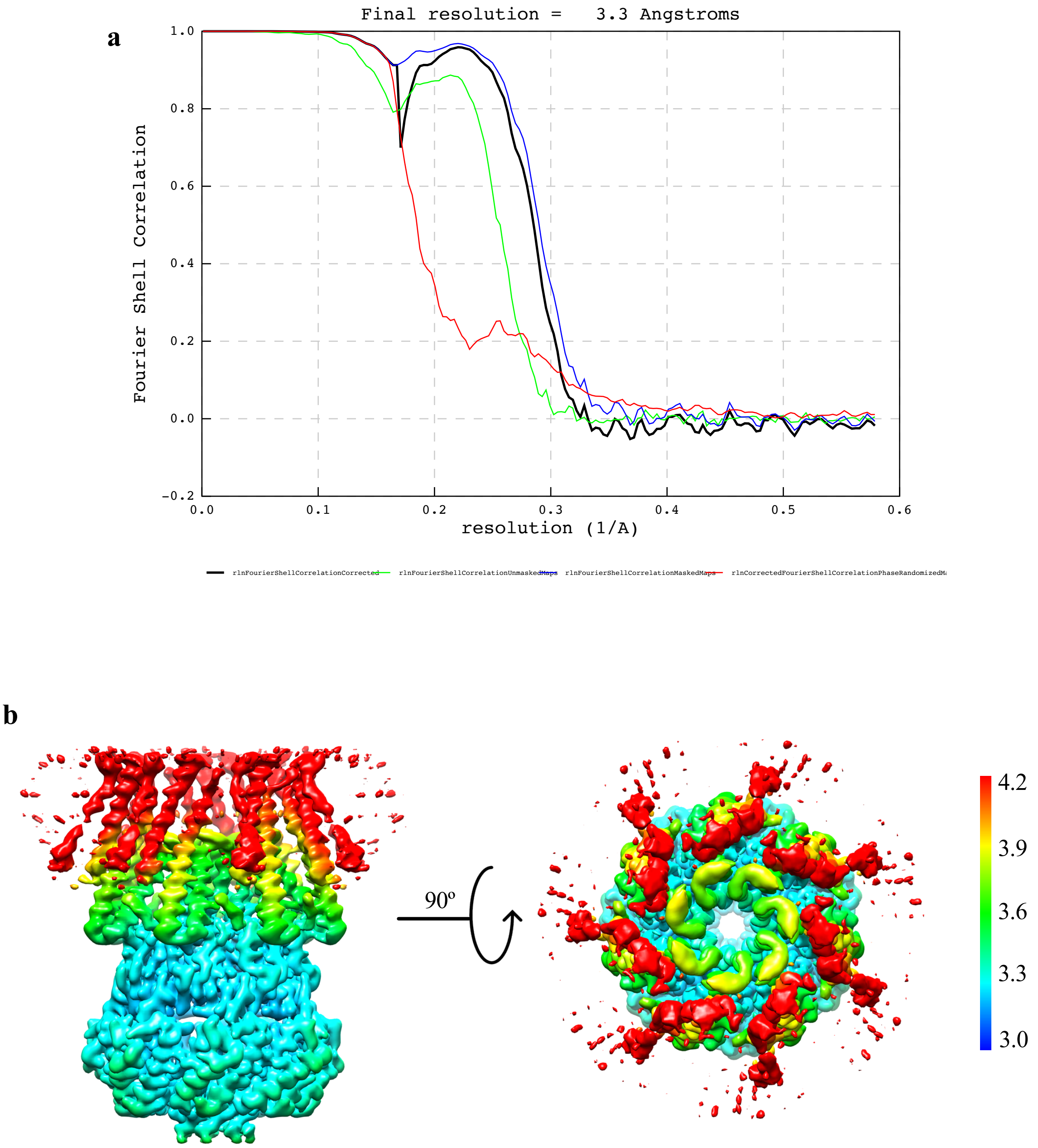

**Figure S5. EM densities of all five TMs.** The density for TM-2 is poor comparing to densities of other TMs, and thus its residues are designated as Ala. The protein is shown in purple, while EM densities are shown as dark gray mesh.

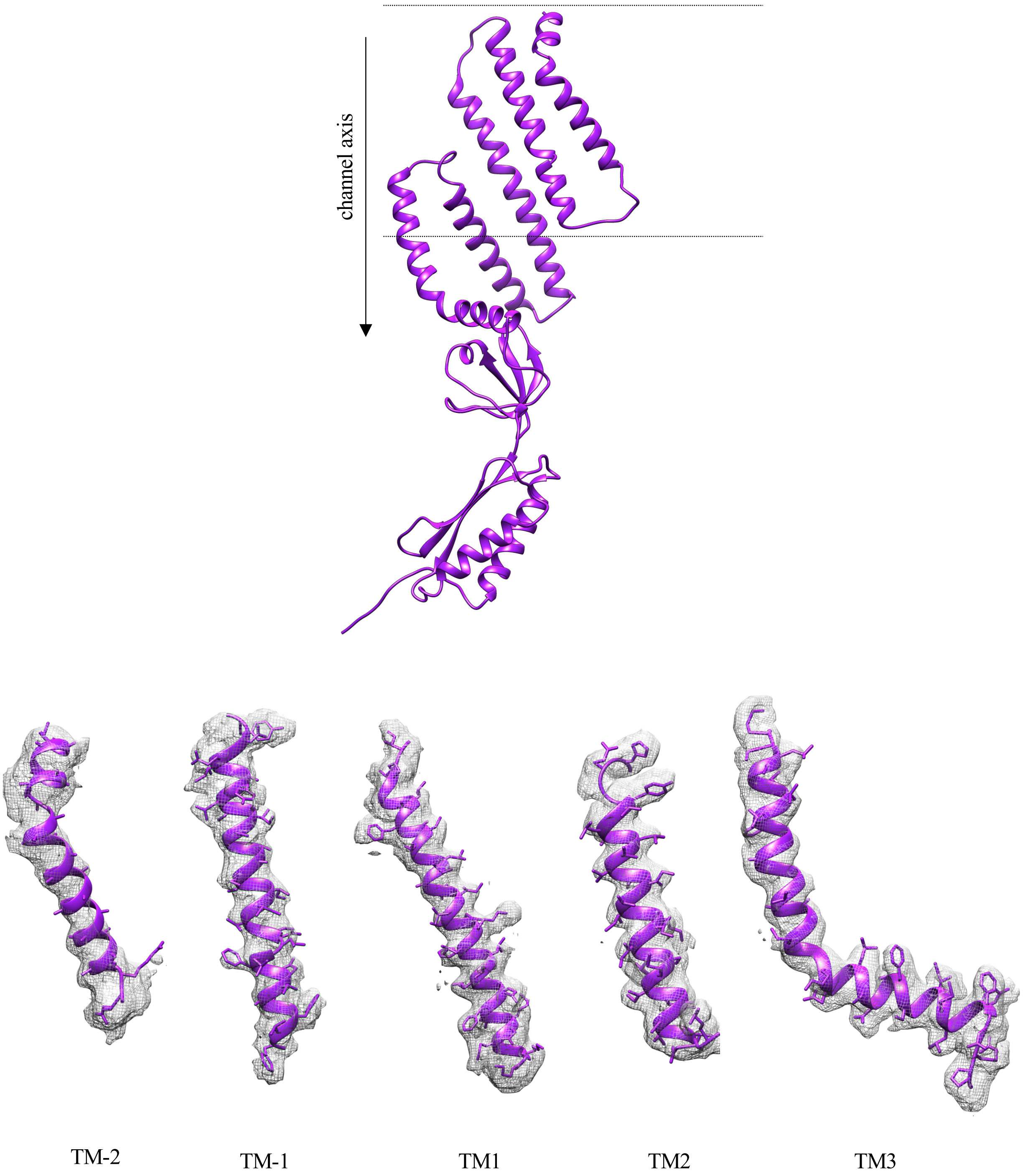

**Figure S6. Mutagenesis studies of residues in the paddle domain. a**, Expression of YnaI-WT and mutants in MS-1106 (DE3) strain detected by Western blot. **b**, Osmotic downshock of MS-1106 (DE3) strain from 500mM NaCl to ~170mM NaCl in LB. The survival rate is calculated by counting colonies. Each experiment is repeated nine times.

a

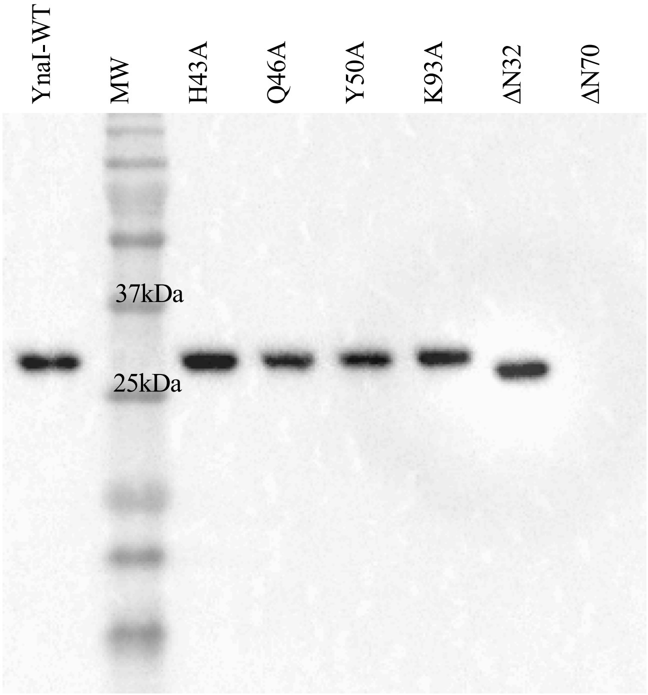


b

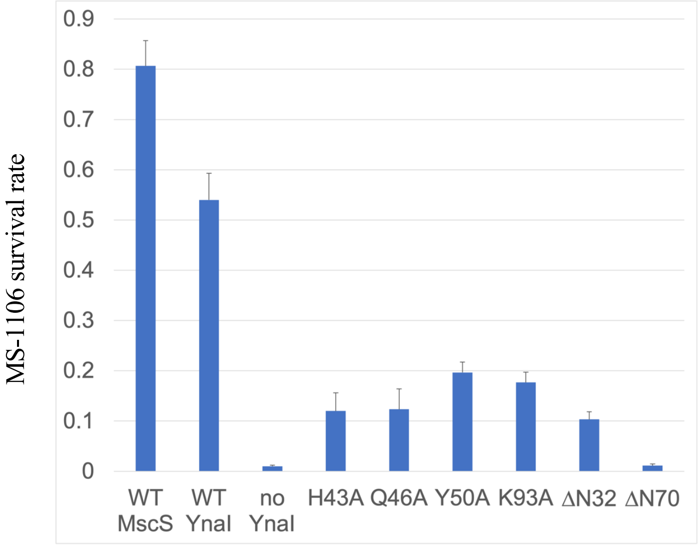

**Figure S7. Co-purified lipids detected by TLC.** The samples loaded from the left to the right are: lane 1, POPA; lane 2, POPS; lane 3, POPC; lane 4, POPE; lane 5 POPG; lane 6, E. coli total lipid extract; lane 7: buffer with 0.01% LMNG; lane 8: purified R120A mutant; lane 9: purified wild type YnaI. Lanes 1~5 loaded 25μg, lane 6 loaded 50μg, lanes 8~9 loaded 300μg.

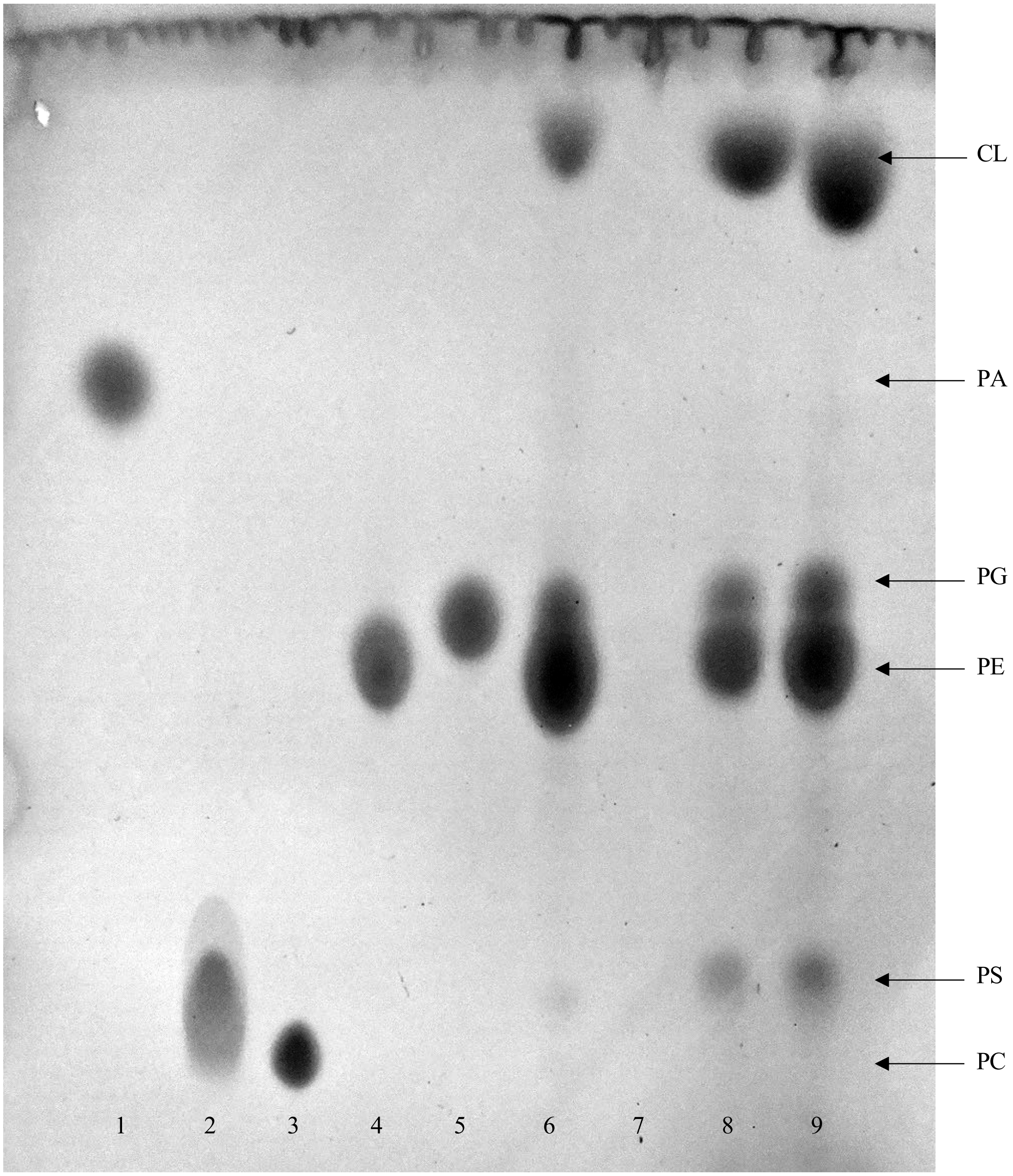

**Figure S8. Pocket comparison between YnaI and MscS. a,** The cone-shaped pockets of MscS in orange. Residues deep in the pocket are shown as ball & stick mode, while residues on the open edge of the pocket are shown as stick. **b,** Comparison of a pocket in YnaI (blue and purple) and MscS (orange). Lipid headgroup interacting residues R120 and Q100 in YnaI structurally correspond well with residues D67 and R59 in MscS when the whole paddle is rotated around the central channel for ~10º.

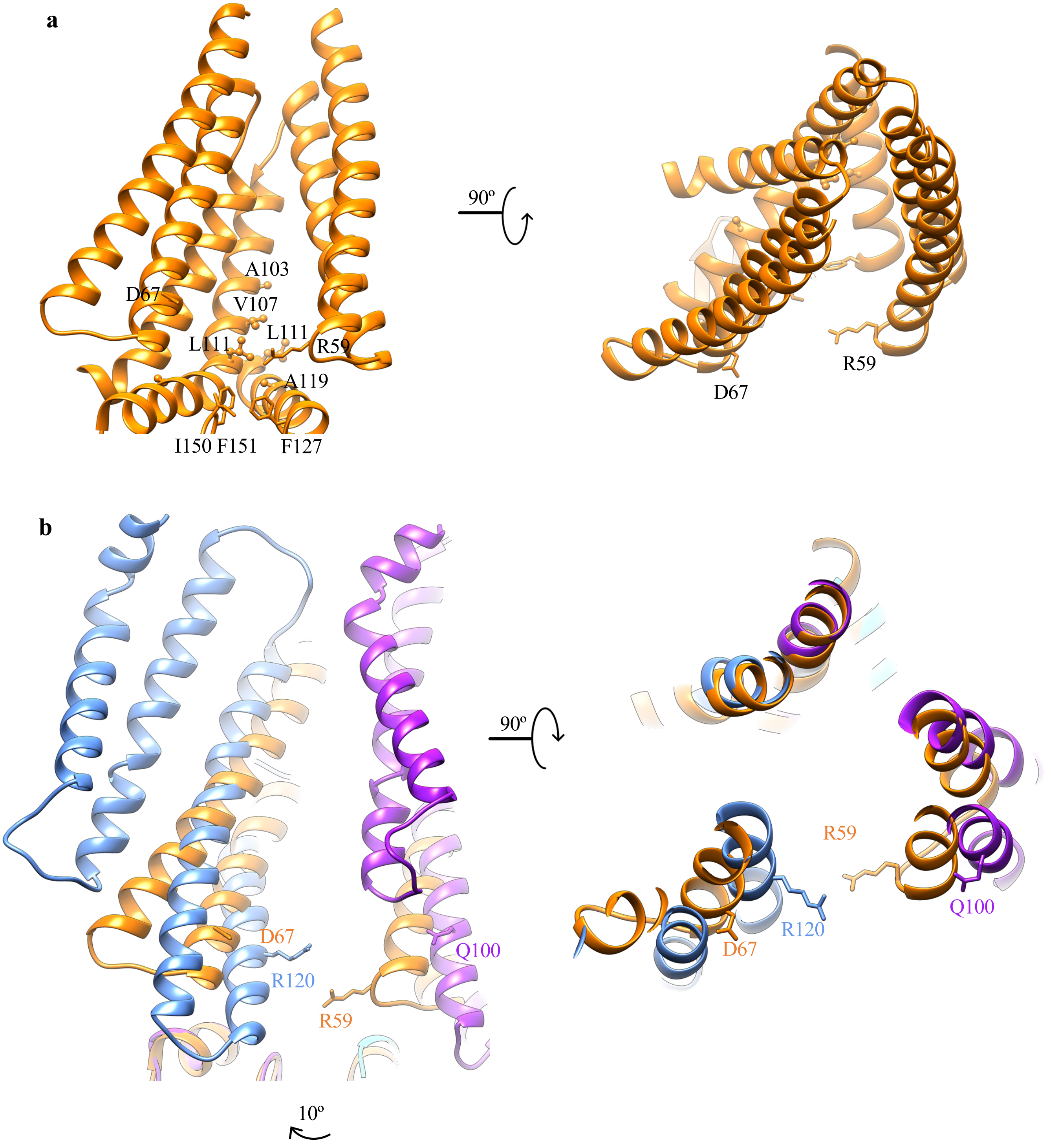

**Figure S9. Mutagenesis studies of residues potentially interacting with lipids. a**, Expression of YnaI-WT and mutants in MS-1106 (DE3) strain detected by Western blot. **b,** *E. coli* cell survival rate under osmotic downshock. The data from 3 independent experiments were analyzed using two-tailed Student's T test. **c**, *E. coli* growth curve with YnaI wild type and mutants at 37ºC.

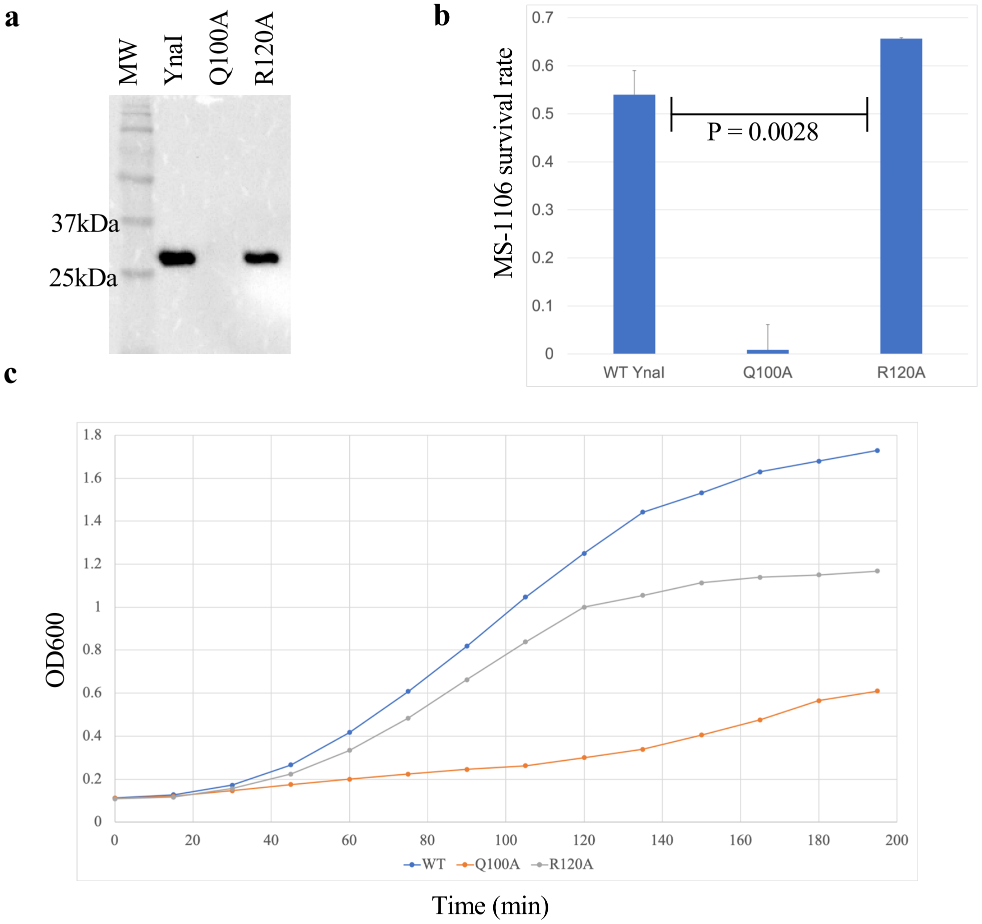

**Figure S10. The side portal of MscS.** Residues F178 and Y135 (equivalent to residues P229 and W184 in YnaI) interact with each other through T-shaped π-π stacking

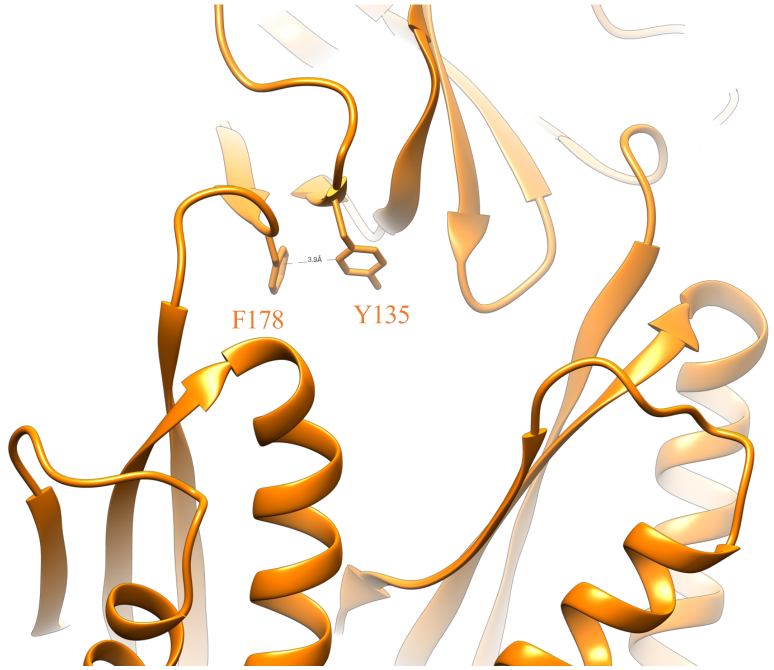

**Figure S11. Coulombic surface charge of MscS vestibules.** Sliced top views of the cytosolic vestibule from **a**. E. *coli* MscS (6PWN) and **b**. T. *tengcongensis* MscS (3T9N). Both are colored by coulombic surface charge computed in Chimera using default settings.

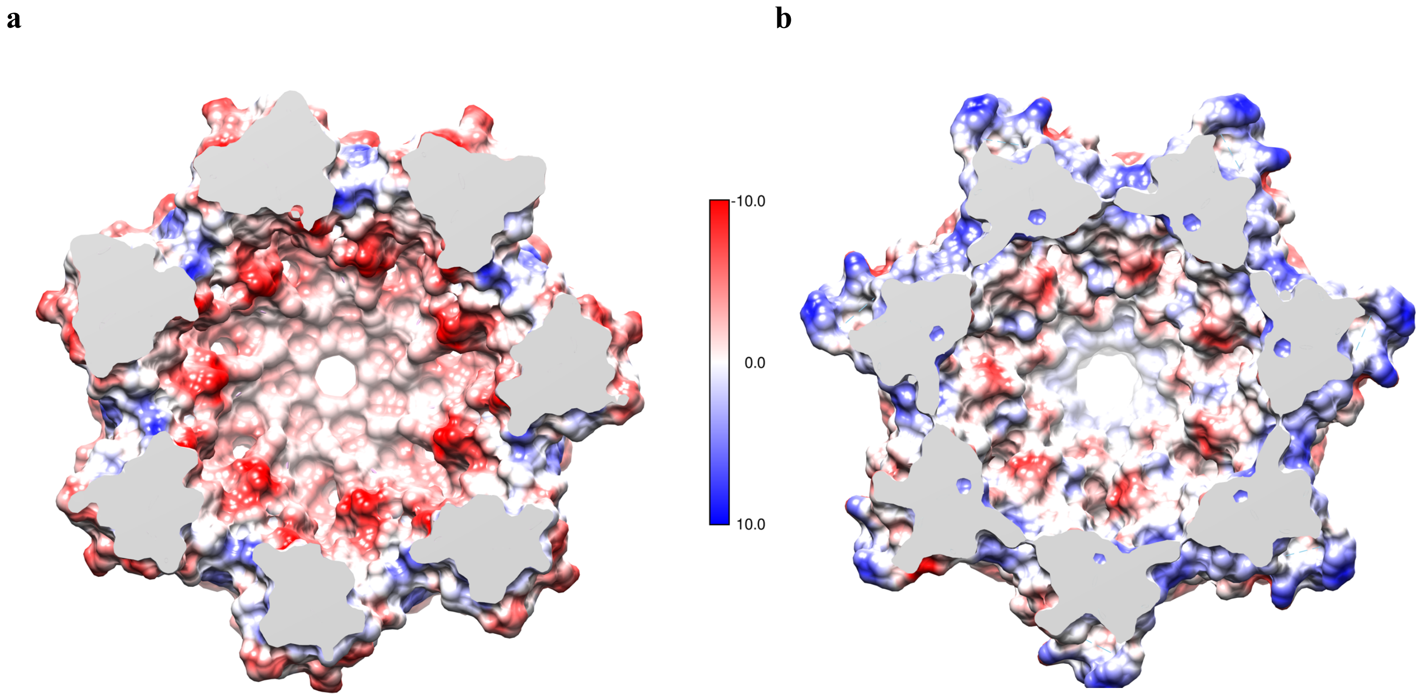

Supplement: Supplementary file 2 — Supplementary Information [file 42003_2021_2122_MOESM2_ESM.pdf]
